# Remarks on Anæsthesia, and the Agents Employed to Produce It

**Published:** 1860-01

**Authors:** George Hayward

**Affiliations:** late Professor of Surgery in the Massachusetts Medical College, Boston, U. S. A.


### ARTICLE XVII.

*Remarks on Anœsthesia, and the agents employed to produce it.* By GEORGE HAYWARD, M. D., late Professor of Surgery in the Massachusetts Medical College, Boston, U. S. A.

THE discovery by which surgical operations can be rendered painless is one of the greatest connected with our profession, second only to that of vaccination. It is a blessing to the human family that cannot be overrated; and having been among the first to make a successful use of it in surgical practice, I thought that a brief sketch of the history of anæsthesia, and some remarks on the comparative value of the agents employed to produce it, would not prove uninteresting.

It was my fortune to perform the first capital operation on a patient rendered insensible by the inhalation of sulphuric ether. This was done on November 7th, 1846, at the Massachusetts General Hospital, Boston. On September 30th, preceding, Dr. Morton, a dentist, administered it to a man, from whom he extracted a tooth without causing pain. Almost immediately after, he requested the late Dr. John C. Warren, who was at that time the acting surgeon at the hospital, to use it at that institution. Dr. Warren consented. It was inhaled by a patient, with partial success, on whom Dr. Warren operated on October 16th. The operation was the removal of a nævus from the face. On the day following, I extirpated a large fatty tumor from the arm of a female, who was made wholly unconscious and insensible by the inhalation of the ether. The operation lasted seven minutes.

At that time Dr. Morton was, I thought, the only person who knew what the anæsthetic agent was. On November 1st, I took charge of the surgical department of the hospital, and in a day or two after Dr. Morton asked me if I were willing to allow him to administer his "composition,"

as he called it, to a female whose limb I was about to remove above the knee. I told him I would not, unless I knew what the article was, and felt confident of the entire safety of its administration. He at once told me that it was rectified sulphuric ether. He allowed me to communicate this to my colleagues, with an understanding that it should not be made known publicly, until he had obtained a patent, for which he had already applied. On the following day the operation was performed, in the presence of more than two hundred spectators.

It rarely falls to the lot of a professional man to be the witness of a scene of more intense interest. The operating room was crowded. Many were obliged to stand. Besides the class of students in attendance on the lectures, numbering more than a hundred, and many of the principal physicians and surgeons of the city and neighborhood, there were present several clergymen, lawyers, and other individuals from the various callings of life. When I entered the theatre, before the patient was brought in, I found it, to my surprise, filled in every part, except the floor on which the table stood, with persons on whose countenances was depicted the almost painful anxiety with which they awaited the result of the experiment they were about to witness. I simply told them that I had decided, with the advice of my colleagues, to allow the patient, on whom I was to operate, to inhale an article which was said to have the power of annulling pain. The patient was then brought in. She was a delicate-looking girl of about 20 years of age, who had suffered for a long time from a scrofulous disease of the knee-joint. It had at length suppurated; there were extensive openings into the cavity of the joint; the cartilages were ulcerated and partly absorbed; the bones carious, and symptoms of hectic fever had already made their appearance. As soon as she was well arranged on the table, I told her that I should let her breathe something which, I hoped, would prevent her from suffering much from the operation, and that she need not be afraid of breathing it freely.

As the ether was at the time administered by means of a large and clumsy instrument, which required to some extent the coöperation of the patient, it was desirable that the amputation should be done as rapidly as possible. Everything, therefore, was arranged with this view. I decided to perform the flap operation. One person was to compress the artery, another to withdraw the flap, a third to hand the instruments, and a fourth to watch the pulse. I grasped the patient's limb with my left hand, and held the amputating knife behind me in my right, carefully concealed from her view. The mouthpiece of the inhaling instrument was then put into her mouth, and she was directed to take long inspirations. After breathing in this way a short time, the nostrils were compressed, so that all the air that went into the lungs must first pass through the machine, and of course be mixed with the vapor of the ether. She breathed with perfect ease and without struggling, and in about three minutes from the time the instrument was put into her mouth, Dr. Morton said, "she is ready." A death-like silence reigned in the room ; no one moved or hardly breathed. I passed the knife directly through the limb, and brought it out as rapidly as I could, and made the upper flap. The patient gave no sign of feeling or consciousness, but looked like one in a deep, quiet sleep. Every other person in the room took a full inspiration that was distinctly audible, and seemed to feel that they could now breathe again. The second flap was then made, the bone sawed, five arteries were tied, and as I was tightening the ligature upon the sixth and last, she groaned, being the first indication of sensibility that had been given. Nothing more was done than to bring the flaps together, cover the stump with cloths dipped in cold water, and apply two or three turns of a roller to keep them in place. Her consciousness soon returned; she was wholly ignorant that the operation had been done. For some time she would not believe it, and said that she had felt nothing till I tied the last artery. The operation lasted a minute and three quarters, not including the time required to tie the arteries. I did it rapidly, though it has been done in less time, because I feared that the insensibility might pass off, and we had no means then, as we have now, of continuing it as long as is necessary.

Patients who have inhaled ether, when its effects are at first passing off, are usually bewildered, not easily controlled, and by no means inclined to do as they are desired. It would be almost impossible to persuade one of them at such a time to breathe through the instrument that was then in use. At present, fortunately, we can keep up the state of anæsthesia as long as we wish, by administering the agent employed for this purpose by means of a sponge. This simple contrivance was first used at the Massachusetts Hospital.

The patient whose case I have just spoken of recovered rapidly from the operation, was in good health when I left home eleven years after, and I have no reason to suppose that she is not so at the present time.

It will be readily believed that a result so successful, and witnessed by so many intelligent persons, made it impossible to doubt the anæsthetic power of the agent employed, and what this was very soon became known. In an almost incredibly short space of time, numerous operations were performed on persons rendered insensible by the inhalation of ether, in various parts of the United States and Europe, and there is hardly a country in christendom in which it has not been thus used to a greater or less extent.

*The Anæsthetic Agents.*—These are sulphuric ether, chloroform, chloric ether, and amylene. The two latter are now rarely used for this purpose, and probably never will be again. Chloric ether is simply a tincture of chloroform. There are two kinds, one the concentrated and the other the chloric ether of commerce. The first is composed of one part of chloroform to nine of alcohol, and in the other ther is one part of chloroform to fifteen of alcohol. It can be prepared by mixing the two ingredients of which it is composed in the proper proportions, and if the alcohol which it contains be evaporated, nothing but chloroform remains. It is evident that it derives its anæsthetic properties from the chloroform, and it is therefore as unsafe as that article; for the alcohol, though it renders it less efficacious, does not make it more harmless.

*Amylene*, the chemical elements of which are equal parts of carbon and hydrogen, has caused death in several instances. There have been so many fatal cases in proportion to the number in which it has been exhibited, that no one hereafter will probably be sufficiently reckless to use it.

*Chloroform* was first employed by Professor Simpson, of Edinburgh, who thought that it possessed "various important advantages" over sulphuric ether. He said that it was more portable, more agreeable to inhale, less exciting, and that it gave a greater control over the patient. That it is more portable and more agreeable to inhale, I admit, but that it is less exciting and a more efficient anæsthetic agent, I deny. But the principal objection to it is, that its inhalation sometimes causes death. Its advocates admit that this has occurred in sixty cases, while others believe that there has been double this number. But be the number what it may, so many have died from its inhalation, that many persons are in favor of abandoning its use altogether. Death produced by it cannot now be attributed in every instance, as it was at first, to the impurity of the article, or to the exhibition of too large an amount, or to the want of skill or judgment in the administrator. There have been several fatal cases lately, where the chloroform was said to be of the purest character, and a small quantity only inhaled, and this, too, in the presence and under the direction of intelligent, well-educated and careful men.

The truth is, that chloroform, when inhaled, acts on the system in a way that is not yet well understood, and may destroy life in spite of the utmost caution. Its effects are sometimes so sudden, that no foresight can prevent a fatal result. Unless some means, therefore, can be discovered that will render its inhalation safe, common prudence and a regard for human life would seem to dictate that it should be no longer used in this way. It is true that the state of unconscious insensibility produced by it is a blessing of countless value to those who are to undergo severe surgical operations, not only by rendering them painless, but at the same time disarming them of their terror. And these are not the only advantages of anæsthesia. It in great measure prevents the shock to the nervous system which not unfrequently defeats the skill of the most expert surgeon, it enables him to operate more deliberately, removes all necessity for haste, which is often the result of the sufferings of the patient, and makes the performance of some operations comparatively easy, which in the ordinary state of the system could hardly be done at all. It is not, therefore, to be wondered at that professional men are reluctant to abandon the use of chloroform, and their unwillingness might be excused if there were not a substitute equally efficacious, as easily administered, and entirely safe. That rectified sulphuric ether is such a one, I have no doubt. I have witnessed its effects on several hundred patients upon whom severe surgical operations were performed, and all of them were rendered motionless, unconscious and insensible. In no instance was there any alarming or serious consequence. It does not act as speedily, perhaps, as chloroform, but in no case were more than eight minutes required to produce complete anæsthesia. It can be effected in much less time when atmospheric air is not allowed to mix freely with the vapor of the ether. This is the method pursued in the hospital at Naples, where no other anæsthetic agent is used ; and I saw a patient undergo a severe surgical operation there without the slightest suffering, who was brought into this state by inhaling ether only a minute and a third ! But when administered thus rapidly, it is apt to produce a distressing cough and sense of suffocation for a moment, and there might be some reason to fear asphyxia from the exclusion to too great an extent of atmospheric air. Professor Polasciano, however, told me that he always gave it in this way, and had never seen any more troublesome symptoms than those I had witnessed in the case just alluded to. These, though distressing to the patient, were of short continuance, and by no means alarming.

There is no doubt in my mind that sulphuric ether should be used as an anæsthetic agent to the entire exclusion of chloroform. It is as efficacious, and I should say without hesitation, after having seen chloroform administered by others in many cases, that ether produces a more complete state of unconscious insensibility. Its effects pass off sooner, and less vomiting, nausea and headache follow its inhalation. It is as easily administered. All that is required for its administration is a bell-shaped sponge with a concavity large enough to cover the nose and mouth. If the patient breathes it gradually, little or no irritation is produced in the larynx and air-passages, there is but little if any cough or sense of suffocation, nor a distressing or unpleasant symptom of any kind.

There may be some persons to whom the odor of ether is offensive and irritating, but they are comparatively few, and even they can be brought under its influence without any very great annoyance.

The quantity of sulphuric ether required to produce anæsthesia depends very much on the manner in which it is administered. If the patient is made to inhale it rapidly, and the atmospheric air is to a great extent excluded, a small amount will be sufficient. From four to eight ounces may be regarded as the average quantity. It is rare to meet with a case in which less than four ounces will be used ; and in protracted operations, in which it is desirable to keep up the state of insensibility for a length of time, I have often given more than eight ounces. The ether should at first be poured on the concave part of the sponge ; one or two ounces will be enough for this purpose. When the inhalation is going on, it is better to pour the ether on the outside of the sponge so as to avoid the necessity of removing it from the face. From half an ounce to an ounce should be used at a time in this way, till anæsthesia is produced. When this takes place, the patient is wholly unconscious, and has no control over the voluntary muscles. He is unable to raise his eyelids when told to do so, and gives no indication of hearing or consciousness, if spoken to in a loud tone. The pulse usually becomes slower than the ordinary standard, though at the beginning of the inhalation it is quicker.

It is, I am confident, a perfectly safe anæsthetic agent. I have not been able to find any well-attested case of death from its inhalation. There may have been such, but they have never come to my knowledge, though I have taken unwearied pains to obtain information on this point.

It has been said, that this may be attributed to the fact that ether is not extensively used, but that if it were, there would probably have been as many fatal cases in proportion from it, as from the inhalation of chloroform. But this statement is not strictly correct; for though ether is not employed as an anæsthetic agent to any extent, if at all, in Great Britain or many parts of Europe, it is used in Lyons, Naples, and is almost the only one that is administered in the principal hospitals of the United States of America, where its now familiar properties were first discovered.

I have given it in several hundred cases, and witnessed its exhibition by others in as many more. I have administered it to infants not three weeks old, and to persons more than threescore years and ten, and have never in a single instance seen an alarming or distressing effect produced by it. On the first introduction of ether into surgical practice, it was not thought safe to allow persons to inhale it in whom there was reason to believe there was any disease of the heart or lungs, or who had any tendency to an affection of the brain and nervous system. But for some years past I have been in the habit of administering it to individuals of this description, and have as yet had no cause to regret it. In such cases I have thought it prudent to have the vapor of the ether inhaled more slowly, so that it may be more diluted with atmospheric air than under ordinary circumstances; of course the patient could not be brought as soon under its influence as when taken in the usual way.

The state of the system which is produced by the inhalation of ether is that of narcotism, similar precisely to what is induced by drinking immoderately wine or other alcoholic liquors. It is a state of intoxication more transient and less dangerous than that from alcôhol. Its effects pass off sooner, because the vapor of the ether begins to escape from the lungs as soon as the patient ceases to inhale it; while alcohol taken into the stomach is carried into the circulation, and mixes with the blood, and in this way acts longer, if not more powerfully on the brain, though its narcotic effect is not so soon produced. It is possible that life might be destroyed by the inhalation of ether, if it be continued uninteruptedly for a great length of time and a great quantity inhaled. Fatal congestion of the brain might thus be produced, as sometimes happens when alcoholic liquor has been taken to excess. But no person of ordinary prudence would administer it in this way. Long before the occurrence of such a result, symptoms of an unequivocal character would indicate the approaching danger.

When death follows the inhalation of chloroform, on the other hand, there is no merciful premonition. The late Dr. Snow, whose experience on the subject was perhaps greater than that of any other person, thought that "*sudden palsy of the heart* is the cause of sudden death from chloroform." In death by asphyxia, the heart beats for some minutes after breathing has ceased; "whereas in some cases of death by chloroform, the breathing has been proved to go on up to the time the pulse stopped and after it."

With the hope that those who may have occasion to employ any anæsthetic agent will at least make a fair trial of *rectified sulphuric ether*, I respectfully submit these remarks to my professional brethren.

[*Brit. and For. Med. & Chir. Review.*

